# Are Vicilins Another Major Class of Legume Lectins?

**DOI:** 10.3390/molecules191220350

**Published:** 2014-12-05

**Authors:** Ana C. Ribeiro, Sara V. Monteiro, Belmira M. Carrapiço, Ricardo B. Ferreira

**Affiliations:** 1 Faculdade de Farmácia de Lisboa, Lisbon University, Lisboa 1649-003, Portugal; E-Mail: acribeiro@ff.ul.pt; 2 Centro de Botânica Aplicada à Agricultura, Instituto Superior de Agronomia, Lisbon University, Lisboa 1349-017, Portugal; 3 CEV, S.A, Zona Industrial de Cantanhede/Biocant Park, Cantanhede 3060-197, Portugal; E-Mail: sam@cev.com.pt; 4 Faculdade de Medicina Veterinária, Lisbon University, Lisboa 1300-477, Portugal; E-Mail: belmira@fmv.utl.pt; 5 Instituto de Tecnologia Química e Biológica, New University of Lisbon, Oeiras 2780-157, Portugal

**Keywords:** globulin, glycosylated receptor, *Lathyrus sativus*, *Lupinus albus*, seed, *Vicia faba*

## Abstract

Legume lectins comprise a structurally related, Ca/Mn-dependent, widespread, abundant and well characterized lectin family when compared to the large number of lectins from other sources described in the literature. Strangely enough, no specific function has been assigned to them aside from a possible role in storage and/or defense. Using a recent and fine-tuned methodology capable of specific lectin identification, β-conglutin, *Vicia faba* vicilin and β-lathyrin, the vicilin storage globulins from *Lupinus albus*, *V. faba* and *Lathyrus sativus*, respectively, were shown to be capable of affinity binding to thoroughly washed erythrocyte membranes and of specific elution with appropriate sugars. Based on this evidence and on sparse data published in the literature, a second family of legume lectins is proposed: the 7S family of storage proteins from leguminous seeds, or family II of legume lectins. These lectins are also structurally related, widespread and well characterized. In addition, they self-aggregate in a Ca/Mg, electrostatic dependent manner and are even more abundant than the family I of legume lectins. Using the same evidence, reserve and defense roles may be attributed to family II of legume lectins.

## 1. Introduction

The seed proteome consist of a complex mixture of proteins with particular properties/functions, such as storage, defense, catalysis, and sugar- or lipid-binding, among many others. Amid these proteins, globulins are the most widely distributed group of storage proteins and the major protein component in the storage tissues of leguminous seeds. They comprise two main families of storage proteins based on their sedimentation coefficients (S_20.w_) [1]: the 7S or vicilin-type globulins (commonly known as vicilins) and the 11S or legumin-type globulins (legumins). The individual globulins from each family exhibit a considerable degree of structural variation (*i.e*., microheterogeneity), mostly derived from post-translational processing. The storage globulins have been studied in detail in legumes, most notably in pea (*Pisum sativum*), soybean (*Glycine max*), lupine (*Lupinus albus*), broad bean (*Vicia faba)* and French bean (*Phaseolus vulgaris*) [2]. Typically, vicilins are sparsely glycosylated, trimeric clusters of combined molecular masses between 140 and 190 kDa [3], which are mobilized during germination and seedling growth [4,5]. As with legumins, the precursor of vicilin subunits contains two cupin domains. The cupin domain is a six-stranded, short β-barrel structure common to several subfamilies of the globulin superfamily of proteins, including the 11S storage proteins, to which vicilins are closely related [2,6,7].

Lectins are proteins of non-immune origin (thus excluding immunoglobulins) which bind in a stable manner (thus excluding enzymes and carbohydrate sensor/transport proteins) to carbohydrates [8]. They comprise a widespread group of proteins which are abundantly present in edible fruits (e.g., banana and tomato), bulbs (e.g., onion, garlic, and shallot), tubers (e.g., potato) and legume seeds (e.g., soybean, peanut, faba bean, chickpea, kidney bean, lupine, and pea) [9,10]. The known biological functions of lectins are multiple and have been reviewed [11,12]. In particular, the role exhibited by lectins in protection from insects (Coleoptera, Homoptera, Diptera and Lepidoptera orders) [12,13,14,15,16,17] and fungi [18,19], as well as their interaction and symbiosis with bacteria [20] have been well documented [21,22].

Legume lectins are probably the most abundant group of the lectin family of proteins. They usually consist of two or four subunits (25–30 kDa), each with one carbohydrate binding site. Interaction with carbohydrates requires tightly bound Ca^2+^ and Mn^2+^ (or another transition metal). Their protein primary structures exhibit remarkable homologies, demonstrating that these proteins have been conserved through-out evolution [23]. The 3-D structures of legume lectins are similar and characterized by a high content of β-sheets and a lack of α-helices. They exhibit several folds corresponding to different carbohydrate binding motifs [24]. The exact role they play in these seeds remains to be elucidated. The observation that they exhibit a pattern of turnover (*i.e*., both synthesis and degradation) which parallels that of legume reserve globulins suggests that legume lectins primordial role may be related to storage.

Vicilins have been reported as being toxic to members of the Coleoptera and Lepidoptera because they inhibit larval development. They also inhibit yeast growth and their binding to yeast cells is mediated by specific binding to fungal cell wall chitin [25]. The deleterious binding of vicilins to chitin has been studied in some detail during the interaction between vicilins and *Callosobruchus maculatus*. Several references in the literature report the capacity of vicilins to bind chitin and chitinous structures in yeast and fungal cell walls [25], as well as in the midgut, ovaries, eggs and feces of the bruchid beetles *C. maculatus* and *Zabrotes subfasciatus* [26,27,28]. This chitin binding capacity explains the fungicide and insecticide activities displayed by many members of the vicilin family of proteins. The present work provides experimental evidence that legume vicilins display lectin activity. This observation, supported by sparse, additional information published in the literature, indicates that legume vicilins comprise another specialized class of abundant lectins in legume seeds, the family II of legume lectins. The evidence provided is based on an improved affinity-binding methodology developed to indentify novel lectins [29]. Using this approach, different vicilins purified from some economically important legumes (*Lupinus albus*, *Vicia faba* and *Lathyrus sativus*), as well as other globulin fractions (*L. albus* γ-conglutin and Blad, the 20 kDa polypeptide which is a stable intermediate of β-conglutin catabolism [30]), were identified as lectins.

## 2. Results and Discussion

Three vicilins, β-conglutin from *Lupinus albus*, β-lathyrin from *Lathyrus sativus* and the vicilin from *Vicia faba* were isolated and used to prepare specific, polyclonal antibodies in rabbits [31,32,33,34]. A fine-tuned methodology developed previously [29], capable of specific lectin identification, was used to assess the lectin properties of vicilins from different legume species. The potential of this methodology was established by confirming the lectin character of γ-conglutin (a minor lupine seed storage globulin, whose role as a reserve protein and classification as a globulin have been questioned [35,36]) and Blad (a 20 kDa polypeptide which accumulates in the cotyledons of 4 to 12-day old *Lupinus* plantlets as a stable breakdown product of β-conglutin catabolism [30,37]). Previous experiments demonstrated the capacity of γ-conglutin and Blad to bind carbohydrates and glycoproteins, but successive attempts failed to show their haemagglutination activity. According to the present day definition of lectin [8], haemagglutination activity is not required. Nevertheless, γ-conglutin and Blad were reported to possess lectin-like activity, rather than being considered as lectins.

### 2.1. Lectin Activity in Different Lupinus albus Protein Fractions

#### 2.1.1. Presence of Lectin Activities in the Albumin Fraction from *Lupinus albus* Seeds

The water-soluble protein (*i.e*., albumin) fraction from *L. albus* dry seed cotyledons was shown to have haemagglutination activity (4 H.U. = 49.5 µg/µL) and was subsequently subjected to sugar-inhibition assays. A panel of thirteen sugars (Table 1) was analyzed and five of them were selected based on their minimal inhibitory concentration (m.i.c.): galactose (m.i.c = 1.7 × 10^−6^ M), melezitose (m.i.c = 3.7 × 10^−3^ M), sialic acid (m.i.c = 3.7 × 10^−3^ M), raffinose (m.i.c = 11.1 × 10^−3^ M) and fucose (m.i.c = 11.1 × 10^−3^ M). These preliminary results indicate that the *Lupinus* albumin fraction contains proteins displaying lectin activity with specificity towards galactose. 

**Table 1:** Sugar inhibition analysis of the haemagglutination activity of *L. albus* albumin fraction.

Sugar (at 0.1 M Initial Concentration)	Sugar Minimal Inhibitory Concentration (m.i.c.) (M)	Maximal Protein Concentration Inhibiting 4 H.U.* (μg/mL)
d-Glucose	0.1	49.5
d-Glucosamine	0.1	49.5
*N*-Acetyl-d-glucosamine	0.1	49.5
d-Galactose	1.7 × 10^−6^	49.5
d-Galactosamine	UD**	-
Sialic acid	3.7 × 10^−3^	49.5
Lactose	UD	-
d-Mannose	0.1	49.5
Raffinose	11.1 × 10^−3^	49.5
l-Fucose	11.1 × 10^−3^	49.5
Melezitose	3.7 × 10^−3^	49.5
α-Methyl-d-glucopyranoside	UD	49.5
Sucrose	3.3 × 10^−2^	49.5

Notes: H.U.* = Haemagglutinant unit is the minimal lectin concentration that causes erythrocyte agglutination; UD** = Undetectable.

#### 2.1.2. γ-Conglutin from *Lupinus albus* Seeds

**Figure 1:**
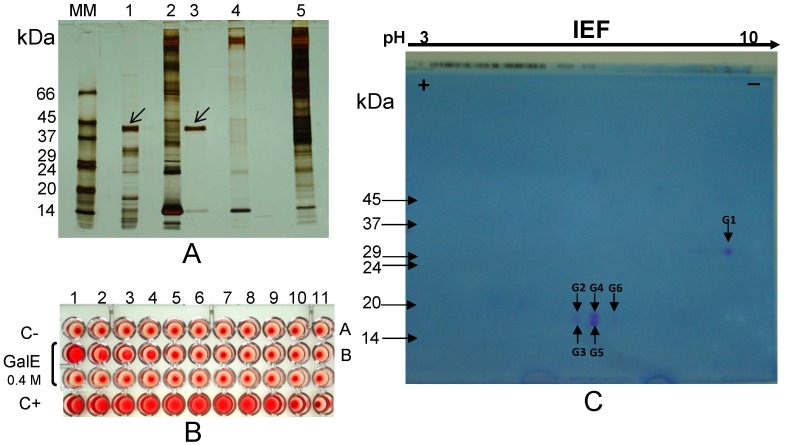
(**A**) NR-SDS-PAGE. The albumin fraction from *L. albus* seeds (lane 1) was incubated with erythrocyte membranes (lane 2). A 42 kDa subunit was eluted with 0.4 M galactose (lane 3) leaving behind a final membrane fraction (lane 5). Control erythrocyte membranes were treated with galactose (lane 4); (**B**) Haemagglutination activity of the galactose eluate (GalE, lane 3) was apparent in the first four wells when compared with the negative control (C-; saline); (**C**) 2-D electrophoretic analysis (2nd dimension performed under reducing conditions) of the 42 kDa subunit reveals the presence of a 30 kDa larger polypeptide (G1) and five isoforms of a smaller (17–18 kDa) polypeptide (G2 to G6). Molecular masses of standards (lane MM) are indicated in kDa. Arrows point to the protein subunit (SDS-PAGE performed under non-reducing conditions) which bound to the erythrocyte membranes.

The albumin fraction from *L. albus* seeds was isolated and incubated with thoroughly washed rabbit erythrocyte membranes (Experimental, Section 3.6), followed by extensive washings and subsequent elution of the bound lectin(s) with galactose (Table 1). Non-reducing, sodium dodecyl sulphate-polyacrylamide gel electrophoresis (NR-SDS-PAGE) of the eluate revealed the presence of water-soluble proteins/protein subunits which bound to glycosylated epitopes on the erythrocyte membranes (Figure 1A). A comparison among lanes 1 (total lupine albumin fraction), 2 (initial erythrocyte membranes), 3 (galactose eluate) and 4 (control eluate) clearly identifies a 42 kDa protein/protein subunit that was specifically eluted from the membranes with galactose (at 0.4 M concentration), which is absent in the control eluate and which is a major polypetide in original albumin fraction. To ensure the subsequent absence of sugars in the galactose eluate, this sample was profusely washed with saline containing 2 mM Ca**^2+^** and 2 mM Mg**^2+^** using dialysis, desalting on Sephadex G-25 PD-10 prepacked columns and ultrafiltration on Centricon membranes (10 kDa cut-off) before haemagglutination activity was determined. A strong haemagglutination activity was detected in the washed galactose eluate (Figure 1B, wells B1 to B4), confirming that the 42 kDa protein/protein subunit exhibits lectin activity.

Two dimensional (2-D)-electrophoresis (2nd dimension performed under reducing conditions) of the 42 kDa eluate revealed that this lectin/lectin subunit exhibits microheterogeneity and comprises two polypeptides linked by disulfide bonds: a 30 kDa basic polypeptide (G1), with a pI ≈ 8.0, and five slightly acidic isoform polypeptides (G2 to G6), with identical molecular masses (17–18 kDa) but different isoelectric points (around pH 6.0) (Figure 1C).

**Figure 2:**
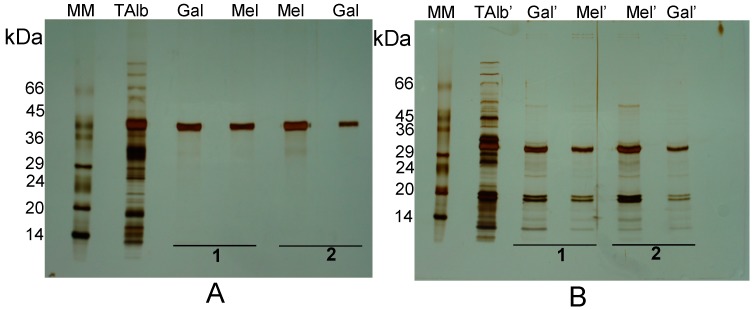
Electrophoretic profile of the galactose-eluted 42 kDa subunit bound to erythrocyte membranes, performed under non-reducing (**A**, NR-SDS-PAGE) and reducing conditions (**B**, R-SDS-PAGE). Two separate but identical sets of erythrocyte membranes were incubated with lupine total albumins (lanes TAlb and TAlb’) and submitted to sequential elution by 0.4 M galactose (elution 1, lanes Gal and Gal’) followed by 0.4 M melezitose (elution 1, lanes Mel and Mel’), or melezitose (elution 2, lanes Mel and Mel’) followed by galactose (elution 2, lanes Gal and Gal’). Molecular masses of standards (MM) are indicated in kDa.

NR-SDS-PAGE (Figure 2A) and R-SDS-PAGE (Figure 2B) analyses confirm the subunit and polypeptide composition of the galactose-eluted 42 kDa lectin/lectin subunit. In these gels, sequential elution from the erythrocyte membranes by galactose followed by melezitose or the other way round produced identical results, clearly indicating that the 42 kDa lectin/lectin subunit displays specificity for the two sugars.

**Figure 3:**
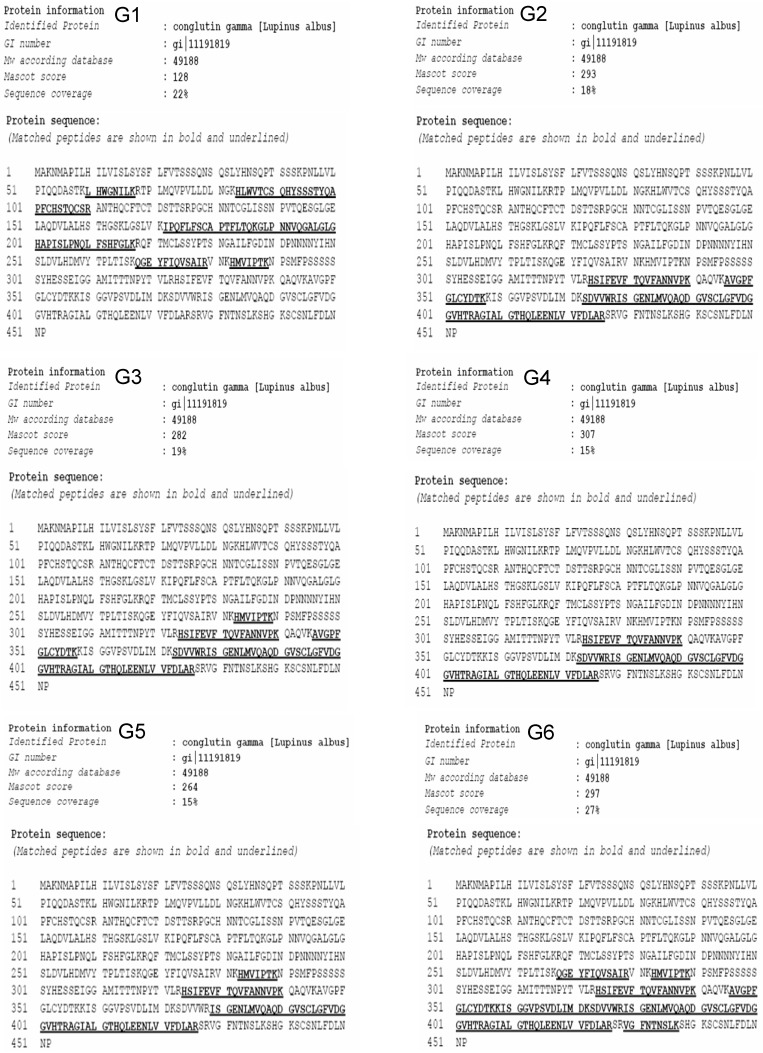
Peptide mass fingerprinting (MALDI-TOF-MS) analysis obtained by LC/MS/MS of the six polypeptide spots (G1 to G6) detected in Figure 1C.

Two-D gel spot (G1 to G6 in Figure 1C) analyses by MALDI-TOF-MS/MS (database MASCOT, version 2.1.03) allowed the identification of the 42 kDa lectin/lectin subunit as γ-conglutin from *L. albus* seeds (Figure 3).

γ-Conglutin pro-polypeptide undergoes post-translational proteolytic cleavage to yield both the large and small polypeptides which compose mature γ-conglutin subunits [38]. Differential proteolytic trimming of the terminal regions, as well as different glycosylation patterns are likely causes for the subunit heterogeneity [38,39,40]. The former may explain the apparently unusual overlapping regions between the large polypeptide (G1) and some (G3, G5 and G6) of the small polypeptide isoforms which compose γ-conglutin subunits (Figure 1C and Figure 3).

In summary, a three step procedure involving (i) extraction of the total albumin fraction; (ii) affinity binding to erythrocyte membranes; and (iii) specific elution with galactose/melezitose allows purification of the lectin/seed storage protein γ-conglutin free from interferents and protein contaminants (Figure 1A and Figure 2A).

#### 2.1.3. β-Conglutin Fraction from 8 DAG *Lupinus albus* Cotyledons (Blad-Containing Oligomer)

β-Conglutin, a vicilin, is the major globulin present in *L. albus* seeds. When analysed by SDS-PAGE, it comprises 10 to 12 major types of subunits (15 to 72 kDa) and a considerable number of minor subunits, with no dissulfide bonds [41]. A more detailed analysis (2-D electrophoresis) reveals the presence of a very large number of distinct polypeptides, indicating a very intense level of post-translational processing of the precursor polypeptide. The majority of these polypeptides may be grouped into five distinct classes, which differ in their mass to charge ratio [37].

β-Conglutin subunits undergo differential catabolism following germination, with different polypeptides being degraded at significantly different rates [42]. A 20 kDa, lectin-like polypeptide termed Blad, which is a stable breakdown product of β-conglutin catabolism, accumulates as part of a 210 kDa oligomer in the cotyledons 4 days after the onset of germination [30]. This oligomer was incubated (4.5 mg) with purified erythrocyte membranes (2 mL), following the methodology described in the Experimental (Section 3.6). The sugar selection for the criterious elution of the Blad-containing oligomer was established based on the fact that the 20 kDa polypeptide is known to interact with immunoglobulins G (IgG) and a variety of other glycoproteins and carbohydrates such as peroxidase, alkaline phosphatase and chitin ([30] and results noy shown). All these polymers contain galactose and *N-*acetyl-**D**-glucosamine in their composition. These sugars were therefore selected to elute Blad from the erythrocyte membranes.

**Figure 4:**
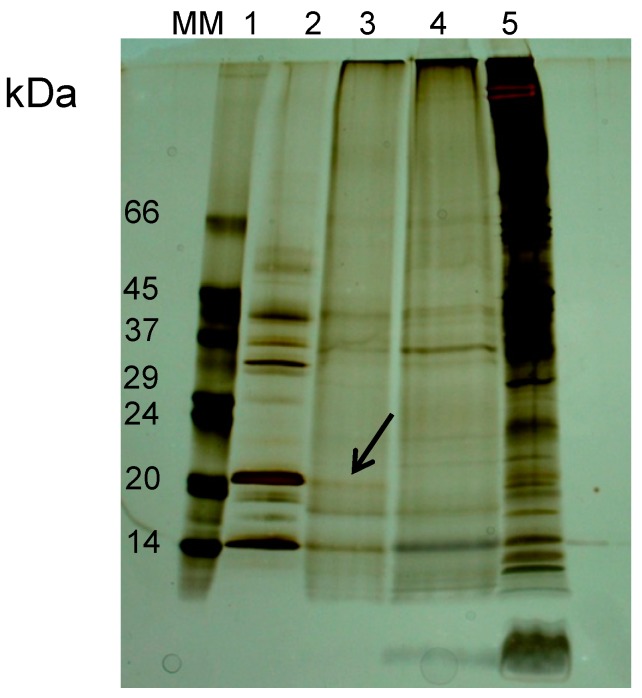
NR-SDS-PAGE electrophoretic profile of Blad elution with 0.4 M galactose (lane 2) from erythrocyte membranes, and comparison with the pure Blad-containing oligomer (lane 1). Galactose elution from a control membrane with no previous protein incubation is presented (lane 3). The final membrane (lane 4) represents the electrophoretic profile remaining in the membrane after Blad elution. Molecular masses of standards (MM) are indicated in kDa. Arrow points to the polypeptide bound to erythrocyte membranes.

Effective elution of Blad from erythrocyte membranes was achieved with 0.4 M galactose (Figure 4). Binding of the 20 kDa polypeptide to the glycosylated receptors on erythrocyte membranes and the corresponding elution with 0.4 M galactose confirms the lectin nature of Blad.

### 2.2. Are Vicilins another Major Class of Legume Lectins?

Vicilins from several legume species have been claimed to bind chitin and in some cases to inhibit insect and fungal growth, as briefly discussed in the introductory section. On the other hand, Blad, a stable breakdown fragment of β-conglutin (the *Lupinus* vicilin) catabolism, is reported to exhibit lectin-like activity [30]. An obvious question is therefore “Can vicilins be considered as another family of legume lectins?” In an attempt to answer this question, total globulins from the dry seeds of three legume species (*Lupinus albus*, *Vicia faba* and *Lathyrus sativus*) were extracted from the respective cotyledons and fractionated by anion-exchange FPLC in order to purify the corresponding vicilins.

#### 2.2.1. The Lectin Nature of *Lupinus albus* Vicilin (β-Conglutin) Subunits

**Figure 5:**
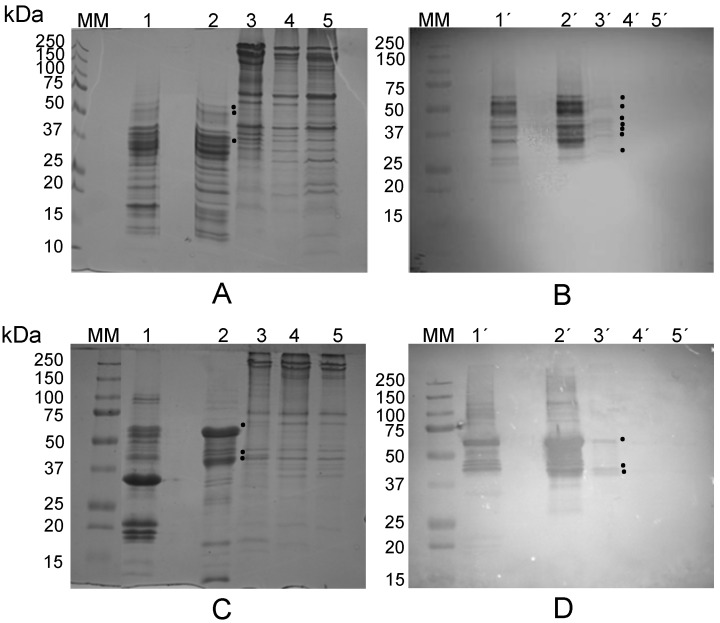
(**A**) Electrophoretic profile (R-SDS-PAGE) of total globulins from *Lupinus albus* seeds (lane 1) versus purified β-conglutin (lane 2). Incubation of erythrocyte membranes with β-conglutin, followed by R-SDS-PAGE and Coomassie Blue R staining revealed that at least three polypeptides bound to the membranes (lane 3). Lanes 4 and 5: initial membrane and final membrane, obtained before incubation and after elution with 0.4 M galactose, respectively; (**B**) An identical gel was blotted onto a nitrocellulose membrane and incubated with anti-β-conglutin antibodies; (**C**,**D**) *Vicia faba* electrophoretic study (R-SDS-PAGE) and immunodetection with anti-*V. faba* vicilin polypeptides, respectively, analogous to (A) and (B). Molecular masses of standards (MM) are indicated in kDa. Black dots indicate polypeptides bound to erythrocyte membranes.

β-Conglutin, the vicilin from *L. albus* seeds, was purified according to Franco *et al.* [43], and subsequently incubated with purified erythrocyte membranes to assess its potential affinity towards membrane glycosylated epitopes. At least three β-conglutin polypeptides (50, 48 and 35 kDa) bound to the membranes were visualized by R-SDS-PAGE (lane 3 in Figure 5A) and seven (56, 51, 46, 43, 40, 36 and 31 kDa) were detected by immunobloting with anti-β-conglutin antibodies (lane 3’ in Figure 5B). As expected, Figure 5B also shows the pattern of β-conglutin polypeptides which were specifically recognized by the anti-β-conglutin antibodies among those comprising the total globulin pattern of polypeptides (lane 1’ compared with lane 2’). The initial membrane and the final membrane, obtained before incubation or after elution of the bound β-conglutin polypeptides with 0.4 M galactose, respectively, did not reveal the present of any polypeptides which could be recognized by the anti-β-conglutin antibodies (lanes 4’ and 5’, respectively).

Previous results reported the affinity of several β-conglutin subunits to glycoproteins, namely immunoglobulins G [30,44], showing that *L. albus* seeds have a pattern of polypeptides analogous to the polypeptides detected in the present work by erythrocyte membrane affinity. These results are also in agreement with the β-conglutin polypeptides which were found to be Blad precursors, comprising class II (30–45 kDa, pI 5.5–9.0) and class III (45–70 kDa, pI 5.0–6.0) of β-conglutin subunits [37], and justify the lectin character of this remarkable polypeptide.

#### 2.2.2. The Lectin Nature of *Vicia faba* Vicilin Subunits

The vicilin from *V. faba* seeds was purified and subsequently incubated with previously isolated erythrocyte membranes as described above. Three vicilin polypeptides (64, 43 and 41 kDa) bound to the membranes and were visualized by R-SDS-PAGE (Figure 5C) and by immunoblotting using anti-*V. faba* vicilin antibodies (Figure 5D). Once again, as expected, Figure 5D also shows the pattern of vicilin polypeptides which were specifically recognized by the anti-vicilin antibodies among those comprising the total globulin pattern of polypeptides (lane 1’ compared to lane 2’). The initial membrane and the final membrane, obtained before incubation and after elution of the bound vicilin polypeptides with 0.4 M galactose, respectively, did not reveal the present of any polypeptides which could be recognized by the anti-vicilin antibodies (lanes 4’ and 5’, respectively). These results indicate that the immunodetected 64, 43 and 41 kDa polypeptides bound to the glycosylated receptors on the erythrocyte membranes.

There is a paucity of scientific information concerning the presence of lectins in *V. faba* seeds. The first report on favin (a seed lectin from *V. faba*) dates back to 1974 [45,46]. The subunit structure of the glucose- and mannose-binding lectin was subsequently characterized by Hemperly and colleagues in 1979 [47]. The molecule is composed of two nonidentical polypeptide chains held together by noncovalent interactions, a smaller α chain (5.6 kDa), and a larger, glycosylated β chain (20 kDa).

The complete amino acid sequence of favin α chain was resolved and shown to be homologous to the α chain of the lectins from lentil and pea, and to residues 72 to 120 of concanavalin A (Con A) from *Canavalia ensiformis* [48]. Hopp and colleagues [49] determined the complete amino acid sequence (182 residues) of favin β chain, and established that the carbohydrate moiety is attached to Asn 168. The β chain is homologous to the β chain of lentil, pea, soybean, peanut and red kidney bean lectins, and is homologous to a portion of the Con A molecule. Together with the sequence of the α-chain previously reported [48], these data completed the primary structure of the lectin. In addition, favin contains a minor component, a β’ chain (18.7 kDa), that closely resembles the β chain, but lacks carbohydrate. The subunit structure of favin closely resembles that of other glucose- and mannose-binding lectins such as those from pea and lentil. Therefore, favin belongs to the widely studied family of legume seed lectins.

A comparative analysis between favin and our results (two *V. faba* vicilin polypeptides with ca. 41 and 43 kDa, which exhibit binding affinity to glycosylated receptors on erythrocyte membranes) shows that our polypeptides differ from those of favin subunits (5 and 20 kDa). The observations that the 41 and 43 kDa polypeptides are vicilin subunits and were recognized by anti-*V. faba* vicilin antibodies further confirm that they are unrelated to favin. Our results support the view that *V. faba* vicilin is a lectin distinct from favin.

#### 2.2.3. The Lectin Nature of *Lathyrus sativus* Vicilin (β-Lathyrin) Subunits

**Figure 6:**
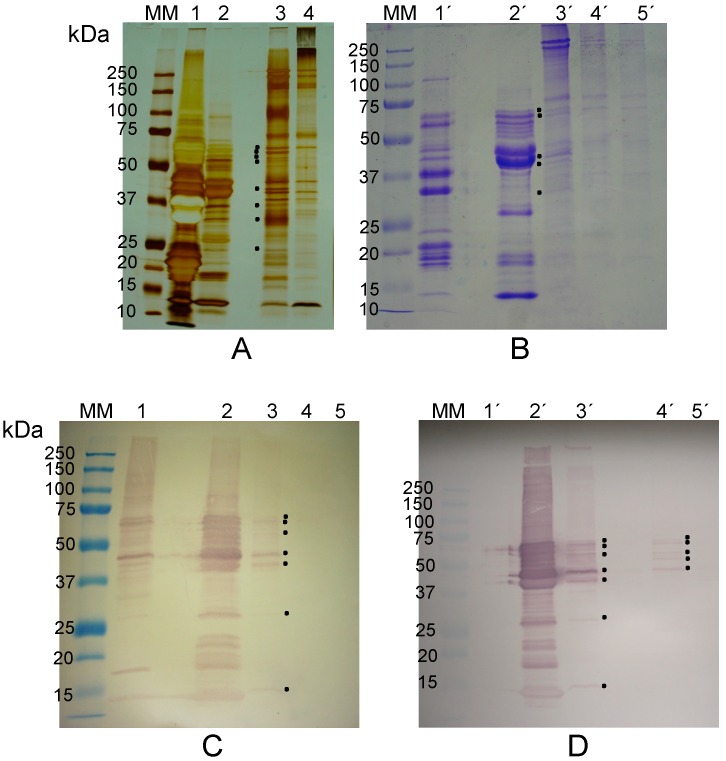
(**A**,**B**) Electrophoretic profile (R-SDS-PAGE; silver nitrate staining in (A), Coomasie Blue R staining in (B)) of total globulins from *Lathyrus sativus* seeds (lanes 1 and 1’) *versus* purified β-lathyrin (lanes 2 and 2’). Incubation of erythrocyte membranes with β-lathyrin, followed by R-SDS-PAGE and Coomassie Blue R staining revealed that at least five polypeptides bound to the membranes (lanes 3 and 3’). Lanes 4, 4’ and 5’: controls—initial membrane (lanes 4 and 4’) and final membrane obtained after incubation with β-lathyrin followed by elution with 0.4 M galactose. (**C**) An identical gel was blotted onto a nitrocellulose membrane and incubated with anti-β-lathyrin antibodies. Lanes 1 to 5 have the same meaning as in (B). (**D**) Immunoblot identical to (C); lanes 2’ and 3’: as in (C); elution of the β-lathyrin polypeptides, bound to glycosylated epitopes of the erythrocyte membrane, by mannose (lane 4’) and galactose (lane 5’). Molecular masses of standards (MM) are indicated in kDa. Black dots indicate polypeptides bound to or eluted from erythrocyte membranes.

*L. sativus* globulins, comprising over 60% of the seed protein, are composed by many different polypeptides covering a wide range of molecular masses [34]. *L. sativus* vicilin, termed β-lathyrin, a minor globulin component, was purified and subsequently incubated with previously isolated erythrocyte membranes as described in the Material and Methods section. β-Lathyrin is composed by several polypeptides (Figure 6A,B) covering a wide range of molecular masses (14 to 66 kDa), in agreement with the data previously reported [34]. After incubation with erythrocyte membranes, at least seven β-lathyrin polypeptides, with masses of 73, 71, 61, 48, 40, 28 and 13 kDa, were found to bind to the membranes by visual detection in silver nitrate stained (Figure 6A) and Coomassie Blue R-250 stained R-SDS-PAGE gels (Figure 6B). Confirmation was achieved by immunodetection using anti-*L. sativus* β-lathyrin antibodies (Figure 6C). Immunodetection of β-lathyrin polypeptides was found in lanes containing *L. sativus* total globulins, purified β-lathyrin and erythrocyte membranes previously incubated with β-lathyrin, but was absent in both the initial and final membranes.

After incubation of β-lathyrin with erythrocyte membranes, these were thoroughly washed with saline containing 2 mM Ca**^2+^** and 2 mM Mg**^2+^**, and then treated with 0.4 M mannose. The resulting eluate was washed with saline, subjected to electrophoresis, blotted onto a nitrocellulose membrane and probed with anti-*L. sativus* β-lathyrin antibodies (Figure 6D). The mannose eluate contains some polypeptides which were previously detected, *i.e.*, 73, 71, 61 and 48 kDa (Figure 6C), and a new polypeptide (57 kDa; Figure 5D). The 57 kDa polypeptide was not visible in Figure 6A,B, most probably because it was not present in sufficient amount. The observation that β-lathyrin 40, 28 and 13 kDa polypeptides were not eluted by 0.4 M mannose suggests the presence of another lectin activity differing in carbohydrate specificity.

### 2.3. The Lectin Nature of Legume Vicilins

The results obtained for the three vicilins under study, β-conglutin from *L. albus*, vicilin from *V. faba* and β-lathyrin from *L. sativus*, are summarized in Table 2. All three vicilins contain polypeptides which bind specifically to carbohydrates present on the erythrocyte membrane matrix, as shown by electrophoretic analyses. These results were confirmed by immunoblotting using specific polyclonal antibodies raised against each of the purified vicilins and showed that the bound polypeptides exhibit molecular masses ranging from 56 to 31 kDa (β-conglutin), 64 to 41 kDa (*V. faba* vicilin) and 73 to 13 kDa (β-lathyrin). A larger number of vicilin polypeptides (*i.e*., seven) capable of binding to erythrocyte membranes were detected in β-conglutin and β-lathyrin, whereas only three polypeptides were detected in *V. faba* vicilin. β-Lathyrin showed the widest range of molecular masses (73 to 13 kDa). Except for *Lupinus*, no lectin-type activity has been previously proposed or suggested for these proteins. For *L. albus* seeds, a lectin-like activity has previously been suggested for the non-vicilin, minor seed storage protein, γ-conglutin [36].

**Table 2:** Vicilin polypeptides from β-conglutin (*L. albus*), vicilin (*V. faba*) and β-lathyrin (*L. sativus*) which bound specifically to glycosylated epitopes present in rabbit erythrocyte membranes.

Legume Species (Seeds)	Immunodetected Polypeptides Specifically Bound to Erythrocyte Membranes (kDa)	Immunodetected Polypeptides from Mannose Eluates (kDa)
*Lupinus albus*	56 ± 2	NT*
	51 ± 2	
	46 ± 2	
	43 ± 2	
	40 ± 2	
	36 ± 2	
	31 ± 2	
*Vicia faba*	64 ± 2	NT
	43 ± 2	
	41 ± 2	
*Lathyrus sativus*	73 ± 2	73 ± 2
	71 ± 2	71 ± 2
	61 ± 2	61 ± 2
	48 ± 2	57 ± 2
	40 ± 2	48 ± 2
	28 ± 2	
	13 ± 2	

Note: NT* = Not tested.

A detailed analysis of Figure 1, Figure 2 and Figure 4, Figure 5 and Figure 6 reveals two potential inconsistencies in the data presented concerning the sugar binding properties of the newly proposed lectins: (i) the low levels of Blad, β-conglutin and *V. faba* vicilin, but not of β-lathyrin and γ-conglutin, which bound to erythrocyte membranes; (ii) the patterns of polypeptides bound to erythrocyte membranes do not seem to match, at least for some cases (*i.e*., β-conglutin) that of the corresponding purified vicilins.

The first potential inconsistency refers to the fact that only a small part of Blad, β-conglutin and *V. faba* vicilin bound to the erythrocyte membranes (see Figure 4 and Figure 5A–D, respectively). The coding capacity of oligosaccharides greatly exceeds those of proteins and nucleic acids. The fact that cells contain many oligosaccharide moieties (as part of *N*- and *O*-linked glycoproteins and of glycolipids) protruding outwards from their plasma membranes and the well-known involvement of these structures in cell-cell interactions and recognition lead to the proposal of the sugar code as the third code of life [11]. The complexity of this code seems to be overwhelming since, unlike the first two codes, no templates seem to exist. If there is a code, one or more decoding tools are required. Lectins fulfill, at least in part, this role. Lectins are therefore expected to have evolved to recognize certain oligosaccharide structures, and the number of these proteins which remain to be discovered is most probably huge. One consequence is that the classification of lectins based on their specificity towards simple sugars, albeit useful, may have little correlation to their biological function, which is expected to be the recognition of precise oligosaccharide structures. This may explain why it is so common to use extremely high sugar concentrations (e.g., 0.3 to 0.5 M) to elute lectins from affinity chromatography gels or from cell membranes. One other difficulty may be to find the correct oligosaccharide structure for which a specific lectin evolved. Given the alleged role in defense attributed to legume lectins in general, it is tempting to speculate that the specific oligosaccharides for which vicilins evolved may be present on the surface of microbial pathogenic agents or, eventually, on another biological source which may no longer exist.

In the past, haemagglutination, or the capacity to agglutinate erythrocytes, was considered as an inherent property of lectins, and was therefore included in the very definition of lectin. For this reason, the term haemagglutinin was commonly employed as a synonym for lectin. In the present work we have used erythrocyte membranes as a source of oligosaccharides to search for lectin activities among legume vicilins. Most certainly, the lectin activity of vicilins did not evolve to recognize specific oligosaccharides present on rabbit erythrocytes. A low level of specificity of the vicilins towards erythrocyte oligosaccharides was to some extent expected and may explain the low level of lectin binding.

In addition, the methodology followed in the present article may have also amplified the effect of the low level specificity. To avoid cross-contamination with β-conglutin or any other vicilin which could have been left from the initial incubation step (legume vicilin with isolated erythrocyte membranes), the membranes precipitated after this incubation step were subsequently thoroughly washed three times followed by centrifugations (8 min at 8000 *g*, 8 min at 14,000 *g* and another 8 min at 14,000 *g*). To further ensure efficient cleaning, these washes were performed with 20 volumes. The resulting pellets, composed of erythrocyte membranes and bound polypeptides, were analysed by SDS-PAGE or treated with appropriate sugars (all at 0.4 M concentration) for elution of the membrane-bound lectins.

The second potential inconsistency may well derive from the former, *i.e*., may result from a low level of lectin binding to erythrocyte membranes. Indeed, a close inspection of Figure 5B (lanes 2’ and 3’) suggests that if the amount of membrane bound polypeptides were increased, the typical pattern of β-conglutin polypeptides (Figure 5B, lane 2) would be obtained. However, due to the particular nature of legume seed storage proteins and their inherent high degree of heterogeneity, it is also possible that not all lectin molecules exhibit the capacity to bind erythrocyte membrane glycoconjugates.

Several reports describe lectins in which the lectin activity is limited to one polypeptide or one subunit of the multipolypeptide or multisubunit protein, respectively. Ricin, for example, is an abundant protein component of *Ricinus communis* seeds that is exquisitely toxic to mammalian cells. Its molecule is composed of one enzymic polypeptide, designated A, which is cytotoxic (it catalyzes the *N*-glycosidic cleavage of a specific adenosine residue from 28S ribosomal RNA), joined by a single disulfide bond to the carbohydrate-binding B polypeptide (the galactose (cell)-binding lectin) [50,51].

Lectins closely related to ricin, designated type 2 RIPs (ribosome-inactivating proteins), have been found in a variety of other plants belonging to diverse taxonomic groups, both in seeds and vegetative tissues. Abrin, from the seeds of *Abrus precatorius*, is similar to ricin. Other examples include modeccin and viscumin, all of which consist of a toxic A polypeptide linked by a disulfide bridge to a carbohydrate-binding B polypeptide [52].

Martínez-Aragón and colleagues [53] isolated five isolectins from *Phaseolus vulgaris* seeds. The isolectins were composed of two distinct types of subunits combined into five different forms of noncovalently bound tetramers exhibiting very different specificities for cell surface receptors. Therefore, each combination was considered to have a different function.

MLL, a lectin with unusual properties both with respect of its specificity and structure, has been isolated from the seeds of *Moluccella laevis*. It consists of three noncovalently associated subunits of 67, 42 and 26 kDa, the largest and smallest of which contain carbohydrate-binding sites [52,54].

We have discovered and studied for the last 23 years a 210 kDa oligomer which is a stable breakdown product of *L. albus* β-conglutin catabolism. All its polypeptide subunits correspond to different length polypeptides of β-conglutin precursor, most of which overlap because they cover the same region of the encoding gene. However, the 20 kDa polypeptide Blad is the only subunit exhibiting lectin activity [30,37] and the only one which seems to bind to erythrocyte membranes (Figure 4, lane 2).

In all lectin examples given above, all protein molecules exhibit lectin activity. However, situations exist in which not all molecules of a given protein with lectin activity exhibit the capacity to bind carbohydrates.

Legume seed storage proteins are characterized by exhibiting a high degree of microheterogeneity. Let us consider β-conglutin from *L. albus*. One-dimensional SDS-PAGE reveals that the mature protein is composed of 10 to 12 major types of subunits, with molecular masses ranging from 15 to 72 kDa, as well as a considerable number of minor constituents [37,41]. However, analysis by two-dimensional electrophoresis allows the detection of a very large number of distinct polypeptides, possibly in the hundreds [37]. This number may be considerably larger taking into account that a change in only a few amino acid residues may not be noticed by one or two-dimensional gel electrophoresis. This extensive level of β-conglutin processing derives at least from differential trimming of β-conglutin precursors and from different levels of glycosylation. Thus, β-conglutin is composed of hundreds of distinct types of subunits, combined into thousands of different forms of noncovalently bound trimers. If only a fraction of β-conglutin subunit types display lectin activity, then many β-conglutin molecules will be unable to bind to erythrocyte membranes.

As evidenced in Figure 1, Figure 2 and Figure 3, the affinity methodology utilized in the present work was successful to scrutinize and confirm the lectin nature of γ-conglutin, as well as that of Blad (Figure 4), confirming the potential of this methodology, which may employ cell membranes from any cell type, supposedly for binding/detecting any type of lectin in any kind of extract [29].

The lectin activities exhibited by β-conglutin, *V. faba* vicilin and β-lathyrin polypeptides have not been reported before. As far as we are aware, the same applies to any other vicilin subunits analysed to date.

Legume seeds have long been considered to host the most abundant family of lectins found in nature, here termed family I of legume lectins. Despite the apparent presence of numerous lectin activities in *L. albus* crude extracts (data not shown), this species constitutes an exception as no member of the family I of legume lectins has been reported after many attempts were made to detect it. Nevertheless, two lectin-like activities have been previously reported in the literature, one relating to γ-conglutin, the other to Blad. However, despite the large number of attempts, no haemagglutination activity could be demonstrated for γ-conglutin or Blad. In this work, haemagglutination activity was demonstrated for γ-conglutin. In addition, the detection of seven β-conglutin polypeptides capable of specific binding to erythrocyte membranes elects β-conglutin and Blad as lectins.

In the case of *V. faba* there seems to be no correspondence whatsoever between the lectins previously described for this species [45,46,47,49] and the vicilin 64, 43 and 41 kDa polypeptides which bound to the erythrocyte membranes. Therefore, *V. faba* vicilin may also be considered as a novel lectin. *L. sativus* seeds are known to contain a 43 kDa lectin which has been isolated by affinity chromatography on Sephadex G-100 as a dimmer composed of 21.5 kDa subunits [48]. Concerning its carbohydrate specificity, the lectin is glucose and mannose specific, and is closely related to concanavalin A and *Lens culinaris* lectin. One other *L. sativus* lectin, a 24 kDa protein with an N-terminal amino acid sequence significantly homologous to the 2S albumin class of seed storage proteins, showed 85% sequence homology to a seed albumin of *Pisum sativum* [55]. None of these *L. sativus* lectins exhibit similarity to the β-lathyrin polypeptides which bound to the erythrocyte membranes. Therefore, β-lathyrin may also be considered as a novel lectin.

We therefore propose the 7S or vicilin family of legume seed storage proteins as a second family (or family II) of legume lectins. As with the family I of legume lectins, these lectins are also structurally related, widespread and well characterized. However, they self-aggregate in a Ca/Mg, electrostatic dependent manner and are even more abundant than family I. Based on the evidence available in the literature, reserve and defense are the main functions attributed to both lectin families.

The studies presented in this work highlight the potential of the affinity methodology used to detect, characterize and purify novel lectins from any source. This methodology was validated and produced positive results for all species/proteins tested so far, which included three legume species (*L. albus*, *V. faba* and *L. sativus*), a characteristic shrub species of the Mediterranean region (*Arbutus unedo* L., strawberry tree), and six pure commercial lectins derived from *Canavalia ensiformis*, *Ricinus communis*, *Triticum vulgaris*, *Tetragonolobus purpurea*, *Arachys hypogaea* and *Dolichos biflorus* [29].

## 3. Experimental Section

### 3.1. Plant Material and Growth Conditions

Dry mature seeds of white lupine *(Lupinus albus* L.) cv. Lublanc were kindly supplied by J.N. Martins (University of Lisbon, Lisbon, Portugal). Dry seeds of broad bean (*Vicia faba* L.) cv. Minor and chickling vetch (*Lathyrus sativus* L.) were obtained in a local Lisbon market. The integuments and embryos were removed and the dry cotyledons were ground to a fine powder using an electric mill (0.2 mm sieve). The resulting meal was used as the source of proteins for the experiments. Cotyledons from eight days old *L. albus* plantlets were obtained as follows: dry mature lupine seeds were immersed under running tap water for 48 h at room temperature. The germinated seeds were then planted in sand and incubated at 25 °C in a 16 h/8 h light/dark cycle under fluorescent lighting. The plantlets were watered as required with water. Eight days after the onset of germination (8 DAG), the seed coats were removed and the intact cotyledons dissected from the axes, frozen in liquid nitrogen and stored at −80 °C until required.

### 3.2. Isolation of Total Albumins and Total Globulins Based on Solubility Criteria

Seeds from *L. albus*, *V. faba* and *L. sativus* were powdered and the resulting meal defatted with *n*-hexane (34 mL/g of flour) for 4 h with agitation and air-dried after decantation of the hexane. Albumins and globulins from *L. albus* and globulins from *V. faba* and *L. sativus* were sequentially extracted and purified using an optimized procedure [43].

Total albumins were extracted by stirring the powder for 4 h at 4 °C in water (pH adjusted to 8.0) containing 10 mM CaCl_2_ and 10 mM MgCl_2_ (34 mL/g of dry mass). The insoluble proteins were removed by centrifugation at 30,000 *g* and 4 °C for 1 h. The supernatant contained the total albumin fraction.

For total globulin extraction, the pellet was resuspended in 100 mM Tris-HCl buffer, pH 7.5, containing 10% (w/v) NaCl, 10 mM ethylenediaminetetraacetic acid (EDTA) and 10 mM ethyleneglycolbis(β-aminoethyl ether)-*N*,*N*,*N*',*N*'-tetraacetic acid (EGTA) (34 mL/g of dry mass), and the suspension was stirred for 4 h at 4 °C. The globulin-containing solution was centrifuged for 1 h at 30,000 *g*, the pellet discarded and the globulins subsequently precipitated by addition of ammonium sulfate (561 g/L). The precipitated globulins were centrifuged at 30,000 *g* for 20 min, resuspended in 50 mM Tris-HCl buffer, pH 7.5, (5.7 mL/g of dry mass), and desalted on PD-10 columns (GE Healthcare Life Sciences; disposable desalting Sephadex G-25 Medium columns, 9.1 mL bed volume) previously equilibrated in the same buffer. All operations were performed at 4 °C.

The presence of CaCl_2_ and MgCl_2_ during the extraction of albumins, and of EDTA and EGTA during the extraction of globulins, increases the extraction efficiency of these proteins and avoids cross-contaminations, as previously shown for a considerable number of legume seeds [43,56].

The cotyledons from 8 DAG seedlings were ground and homogeneized with a mortar and pestle in water (pH adjusted to 8.0) containing 10 mM CaCl_2_ and 10 mM MgCl_2_ (2 mL/g fresh weight). The homogenate was incubated at 4 °C for 30 min with agitation, filtered through cheesecloth and centrifuged at 30,000 *g* for 1 h at 4 °C. The precipitate was suspended in the globulin solubilising buffer (2 mL/g fresh weight; 100 mM Tris-HCl buffer, pH 7.5, containing 10% (w/v) NaCl, 10 mM EDTA and 10 mM EGTA) and agitated during 30 min at 4 °C. The globulin containing solution was centrifuged for 1 h at 30,000 *g* and 4 °C and the resulting supernatant desalted on PD-10 columns previously equilibrated in 50 mM Tris-HCl buffer, pH 7.5.

### 3.3. Purification of Individual Globulins

Total seed globulins were fractionated by FPLC anion-exchange chromatography on a Q-Sepharose (Ø = 1 cm; h = 8 cm) column (GE Healthcare, Uppsala, Sweden) as described before for *L. albus* conglutins [43]. The column was equilibrated in 50 mM Tris-HCl buffer, pH 7.5. Three 2 mL aliquots were loaded into the column. Protein fractionation took place during 80 min with a flow rate of 1.5 mL/min, and the bound proteins were eluted with a linear gradient (0–1 M) of NaCl. One mL fractions were collected. Individual globulins, namely β- and γ-conglutins from *L. albus* dry seeds and the vicilins from *V. faba* and *L. sativus* dry seeds were purified by this method.

### 3.4. Purification of the Native Oligomer Containing Blad

After isolation of the total globulin fraction from 8 DAG *L. albus* cotyledons, followed by precipitation with ammonium sulfate and desalting, the protein corresponding to β-conglutin was purified as described above, with the following exceptions [37]: (i) FPLC anion exchange chromatography on the Q-Sepharose column equilibrated in 20 mM Tris-HCl buffer, pH 7.5; (ii) the fraction containing the 20-kDa polypeptide eluted between 0.25 and 0.35 M NaCl; (iii) the native oligomer containing Blad was further purified by FPLC gel filtration chromatography on a Superose 12 column.

### 3.5. Haemagglutination Assays

Preparation of erythrocytes from rabbit blood, haemagglutination activity assays, and sugar inhibition of haemagglutination activity assays were performed as described before [29].

### 3.6. Affinity Binding of Lectins to Isolated Erythrocyte Membranes

Isolation of erythrocyte membranes was performed according to Ribeiro *et al*. [29]. The membranes isolated from rabbit erythrocytes were used as targets to bind lectins from the five protein fractions under study: the albumin fraction and purified β-conglutin (vicilin) from *L. albus* seeds, the β-conglutin fraction (the Blad-containing oligomer) from 8 DAG *L. albus* plantlets, and the purified vicilins from *L. sativus* and *V. faba* seeds. Ten mg of *L. albus* albumin fraction, 4.5 mg of the Blad-containing oligomer, 10 mg of *L. albus* vicilin, 6 mg of *L. sativus* vicilin and 2.5 mg of *V. faba* vicilin were incubated with a 2 mL pellet of erythrocyte membranes. Each mixture was stirred for 30 min at 25 °C and washed three times followed by centrifugation (8 min at 8000 *g*, 8 min at 14,000 *g* and 8 min more at 14,000 *g*). The resulting pellets, composed of erythrocyte membranes and bound polypeptides, were analysed by SDS-PAGE or treated with appropriate sugars previously selected in the haemagglutination activity inhibition assays (all at 0.4 M concentration) for specific elution of the membrane-bound lectins; γ-conglutin (galactose and melezitose), β-conglutin (galactose) and the Blad-containing oligomer (galactose) from *L. albus*, vicilin (galactose) from *V. faba*, and vicilin (mannose) from *L. sativus*. Appropriate erythrocyte membrane controls were prepared for each sugar in which the membranes were incubated with saline instead of the protein fractions. Sugar-induced elution of the bound proteins took place during 30 min at 25 °C with agitation, followed by centrifugation at 43,000 *g* for 15 min. The supernatants containing the eluted lectins were desalted and extensively dialysed against saline containing 2 mM CaCl_2_ and 2 mM MgCl_2_ for complete removal of the sugar used in the lectin elution step. Confirmation of the lectin presence was achieved by assaying some eluates for haemagglutination activity.

### 3.7. Production of Polyclonal Antibodies

Polyclonal antibodies were produced in rabbits against β-conglutin from *L. albus*, vicilin from *V. faba* and β-lathyrin from *L. sativus* as described before [44]. 

### 3.8. Electrophoresis and Western Blotting

The discontinuous buffer system described by Laemmli was used for polyacrylamide gel electrophoresis (PAGE) [57]. Electrophoresis was performed in slab gels, 16 cm × 18 cm × 0.75 mm. Two types of electrophoresis were used, namely, nonreducing sodium dodecyl sulfate-PAGE (NR-SDS-PAGE) and reducing SDS-PAGE (R-SDS-PAGE), following the methodology previously described [58]. Before electrophoresis, all protein samples were boiled for 3 min in the presence of sample buffer containing 2% (w/v) SDS (NR-SDS-PAGE) or 2% (w/v) SDS and 0.1 M β-mercaptoethanol (R-SDS-PAGE).

Proteins separated by R-SDS-PAGE were transferred onto a nitrocellulose membrane (previously incubated for 15 min in transfer buffer: 39 mM Trizma base, 48 mM glycine, 0.1% (w/v) SDS, 20% (v/v) methanol, pH 8.3) at 15 V for 1.15 h using a semidry transfer unit (Bio-Rad). After protein transfer, the polypeptides on the membrane were fixed for 15 min with a solution containing 10% (v/v) acetic acid and 25% (v/v) 2-propanol. Total polypeptides in the membrane were visualized with Ponceau S.

Two-dimensional (2-D) electrophoresis of the major 42 kDa subunit present in the albumin fraction from *L. albus* cotyledons was carried out as follows. The first dimension, isoelectric focusing (IEF), was performed using the IPGphor system (GE Healthcare). Immobiline Drystrip gel strips (IPG strips) (13 cm, pH 3 to 10) were obtained from GE Healthcare. IPG strips were rehydrated with 250 µL of 0.5% (v/v) IPG-buffer pH 3–10, 7 M urea, 2 M thiourea, 2% (v/v) NP-40, 1% (w/v) dithiothreitol and protein sample in the IPGphor strip holders. The program utilized for IEF included the following steps: rehydration 30 V.h, 12 h; step 1—250 V.h, 1 h; step 2—500 V.h, 2 h; step 3—1000 V.h, 2 h; step 4—2500 V.h, 3.5 h; step 5 (gradient)—8000 V.h, 1 h; and step 6—8000 V during 25 min. After focusing, the gel strips were immediately frozen at −80 °C.

The second dimension, R-SDS-PAGE, was performed as described above except that the gel contained only the separating gel. After IEF, the gel strips were thawed and equilibrated for 15 min, with agitation, in 50 mM Tris-HCl buffer, pH 8.8, containing 6 M urea, 30% (v/v) glycerol, 2% (w/v) SDS and 1% (w/v) dithiothreitol. The strips were subsequently equilibrated for another 15 min, with agitation, in a similar solution that contained 2.5% (w/v) iodoacetamide instead of dithiothreitol, placed on top of a 15% (w/v) acrylamide SDS-PAGE gel, sealed with 0.7% (w/v) agarose (containing 0.002% (w/v) bromophenol blue) and electrophoresed (220 V, 15 mA for 15 min followed by 220 V, 30 mA). After electrophoresis the gel was stained with Coomassie Blue G-250. The visualized spots were picked-up for polypeptide sequencing by MALDI-TOF or LC MS-MS analysis.

### 3.9. Immunoblotting

When appropriate, the vicilins purified from *L. albus*, *V. faba* and *L. sativus*, as well as proteins bound to erythrocyte membranes and eluted with specific sugars, were separated by R-SDS-PAGE and blotted onto nitrocellulose membranes for immunodetection with polyclonal antibodies previously prepared against purified samples of the same proteins. Appropriate controls of the proteins from *L. albus*, *V. faba*, *L. sativus* and rabbit erythrocyte membranes were tested. Control samples, consisting of non-treated erythrocyte membranes and eluates from non-treated erythrocyte membranes were prepared in the same manner. After polypeptide transfer onto nitrocellulose, the membranes were fixed as described above, then washed for 10 min in PBST (20 mM phosphate buffer, 140 mM NaCl, 20 mM KCl, 0.05% v/v Tween 20, pH 7.4) prior to blocking for 1 h in 1% (w/v) dry skimmed milk in PBST. Antibodies specific for each legume vicilin were used at 1:500 (*L. albus*), 1:1500 (*V. faba*) and 1:100 (*L. sativus*) dilutions in PBST, followed an incubation of 1 h at room temperature with gentle shaking. Membranes were washed in PBST twice for 5 min before addition of the secondary antibody specific for rabbit IgGs (A3812, Sigma-Aldrich, St. Louis, MO, USA) conjugated to alkaline phosphatase, and used at 1:9000 dilution in PBST. Detection of the immunocomplex was achieved by hydrolysis of 5-bromo-4-chloro-3-indolyl phosphate (BCIP). This reaction was stopped by addition of water. The blot was rinsed twice for 30 s in water and allowed to dry at room temperature.

### 3.10. Polypeptide Sequencing

The purified polypeptides to be sequenced were visualized on SDS-PAGE gels by Coomassie Blue G-250 staining and the spots sliced, washed with water, and subjected to sequencing by mass spectrometry (peptide mass fingerprinting) at Eurosequence (www.eurosequence.nl).

### 3.11. General Assays

Protein concentration was measured by a modification of the Lowry method [59] or by the Bradford method [60]. Bovine serum albumin was used as the standard. SDS-PAGE and 2-D gels were stained by Coomassie Brillant Blue R 250, Coomassie Brillant Blue G-250 [61] or silver stained [62].

## 4. Conclusions

From the quantitative point of view, three well established, prominent protein families are found in legume seeds: (i) the vicilins and (ii) the legumins, two structurally-related, widespread, extremely abundant and well characterized globulin families, which self-aggregate in a Ca/Mg, electrostatic dependent manner and whose major role in seeds is directed to storage; (iii) legume lectins (or family I of legume lectins), comprising a family of structurally related, Ca/Mn-dependent, widespread, abundant and well characterized proteins, to which no specific function has been assigned. Their abundance and pattern of turnover, with both synthesis and degradation paralleling those of the legume reserve globulins suggest that family I of legume lectins may play a role related to storage.

The data presented in the present work and on sparse data published in the literature fit well with the proposal that vicilins comprise a second family of legume lectins, the family II of legume lectins.

Defense is one of the roles generally attributed to lectins. The recent discovery of lectins in the plant xylome strongly points to a role in defense. It is tempting to speculate that binding of lectins to carbohydrate residues located in the external surface of pathogenic microorganisms may depolarize their cells and inhibit their growth, much in the same way as tannins do when they bind to exoproteins. In addition, the observation that many legume reserve proteins are glycosylated (e.g., in *Lupinus* seeds) may also confer legume seed lectins a role in storage protein efficient packing inside protein storage vacuoles.

Vicilins have been reported as being toxic to the Coleoptera and Lepidoptera members because they inhibit larval development. Vicilin binding to chitin and chitinous structures such as those composing yeast and fungal cell walls and those in the midgut, ovaries, eggs and feces of the bruchid beetles *Callosobruchus maculatus* and *Zabrotes subfasciatus* explains the fungicide and insecticide activities displayed by many members of the vicilin family of proteins. Reserve and defense roles may therefore be attributed to both family I and family II of legume lectins.
